# Assessment of adherence to the CONSORT statement for quality of reports on randomized controlled trial abstracts from four high-impact general medical journals

**DOI:** 10.1186/1745-6215-13-77

**Published:** 2012-06-07

**Authors:** Saurav Ghimire, Eunjung Kyung, Wonku Kang, Eunyoung Kim

**Affiliations:** 1 Department of Clinical Pharmacy, College of Pharmacy, Chungnam National University, 99, Dehak-ro, Yuseong-gu, Daejeon, 305-764, South Korea; 2 College of Pharmacy, Yeungnam University, Kyoungbuk, 712-749, South Korea

**Keywords:** Randomized controlled trials, CONSORT for abstracts, Quality of reports, General medical journals

## Abstract

**Background:**

The extended Consolidated Standards of Reporting Trials (CONSORT) Statement for Abstracts was developed to improve the quality of reports of randomized controlled trials (RCTs) because readers often base their assessment of a trial solely on the abstract. To date, few data exist regarding whether it has achieved this goal. We evaluated the extent of adherence to the CONSORT for Abstract statement for quality of reports on RCT abstracts by four high-impact general medical journals.

**Methods:**

A descriptive analysis of published RCT abstracts in The New England Journal of Medicine (NEJM), The Lancet, The Journal of American Medical Association (JAMA), and the British Medical Journal (BMJ) in the year 2010 was conducted by two reviewers, independently extracting data from a MEDLINE/PubMed search.

**Results:**

We identified 271 potential RCT abstracts meeting our inclusion criteria. More than half of the abstracts identified the study as randomized in the title (58.7%; 159/271), reported the specific objective/hypothesis (72.7%; 197/271), described participant eligibility criteria with settings for data collection (60.9%; 165/271), detailed the interventions for both groups (90.8%; 246/271), and clearly defined the primary outcome (94.8%; 257/271). However, the methodological quality domains were inadequately reported: allocation concealment (11.8%; 32/271) and details of blinding (21.0%; 57/271). Reporting the primary outcome results for each group was done in 84.1% (228/271). Almost all of the abstracts reported trial registration (99.3%; 269/271), whereas reports of funding and of harm or side effects from the interventions were found in only 47.6% (129/271) and 42.8% (116/271) of the abstracts, respectively.

**Conclusions:**

These findings show inconsistencies and non-adherence to the CONSORT for abstract guidelines, especially in the methodological quality domains. Improvements in the quality of RCT reports can be expected by adhering to existing standards and guidelines as expressed by the CONSORT group.

## Background

Randomized controlled trials (RCT) are considered evidence of the highest grade in the hierarchy of research designs [1]. Because these reports can have a powerful and immediate impact on patient care, accurate and complete reporting concerning the design, conduct, analysis, and generalizability of the trial should be conveyed [2]. Interpretation of RCT results becomes difficult, if not impossible, with inadequate reports causing biased results to receive false reliability [3]. Thus, adequate reporting is essential for the reader in evaluating how a clinical trial was conducted and in judging its validity [4].

Regarding the methodological details in describing medical research, focusing on RCTs [5], the most prominent guideline, the Consolidated Standards of Reporting Trials (CONSORT), was published [2] and has been updated regularly [3,6,7]. Because abstracts are the only substantive portion of a paper that many readers read, authors need to be vigilant that they precisely reflect the content of the article. Thus, the International Committee of Medical Journal Editors (ICMJE) emphasized that articles reporting clinical trials should contain abstracts that include CONSORT items identified as essential [8]. Preliminary appraisals suggest that the use of CONSORT items is associated with improvements in the quality of RCTs being reported [9]. Nevertheless, inconsistent [10] and suboptimal [11,12] results have also been found. With respect to the recent recommendations from CONSORT for Abstracts [7], the reporting quality of RCT abstracts published in four major general medical journals, The Journal of American Medical Association (JAMA), British Medical Journal (BMJ), The Lancet, and The New England Journal of Medicine (NEJM) in 2006 was found to be suboptimal [12]. This evaluation will offer an update as to whether CONSORT for Abstracts improves the quality of reports, given the suboptimal reporting by these highly esteemed journals.

Adherence to CONSORT requires continual appraisal for improving the precision and transparency of published trials. Consequently, in an effort to promote quality reports of RCT abstracts, we evaluated the extent of adherence to the CONSORT for Abstracts statement for quality of reports in four high-impact general medical journals published during the year 2010.

## Methods

### Data sources

We selected four high-impact general medical journals: JAMA, BMJ, The Lancet, and NEJM. These journals endorsed the CONSORT in 1996, except the NEJM, which did so in 2005 [10]. Under the article submission instructions for the authors, the two journals, BMJ and The Lancet, clearly recommended following the CONSORT for Abstracts guidelines, whereas JAMA and NEJM referred to the ICMJE’s ‘Uniform Requirements for Manuscripts Submitted to Biomedical Journals’ where the abstract section is required to prepare in accordance with the CONSORT for Abstracts guidelines [13-16]. We conducted a MEDLINE/PubMed search to identify all RCTs published between January and December 2010 with the following search strategy: “Lancet” [Jour] OR “JAMA”[Jour] OR “The New England journal of Medicine”[Jour] OR “BMJ”[Jour] AND (Randomized Controlled Trial[ptyp] AND medline[sb] AND (“2010/01/01”[PDAT] : “2010/12/31”[PDAT])).

### Study selection

RCT abstracts of preventive and therapeutic interventions were selected. We included abstracts in which the allocation of participants to interventions was described by the words random, randomly allocated, randomized, or randomization. However, the abstracts of other study designs were excluded: observational studies, economic analyses on RCTs, quasi-randomized trials, cluster randomized trials, diagnostic or screening tests, follow-up studies of previously reported RCTs, editorials, and letters. The modified CONSORT extension for cluster randomized trials [17] and separate Standards for the Reporting of Diagnostic Accuracy (the STARD) statement for reporting studies of diagnostic tests [18] prevented us from including these studies.

### Data extraction

Two reviewers (SG and EjK) underwent training in evaluating RCTs using the CONSORT for Abstracts [7] and begun the data extraction. A ‘yes’ or ‘no’ answer was assigned for each item indicating whether the author had reported it. A pilot study was performed with randomly selected abstracts to assess inter-observer agreement using the kappa value [19] and to resolve any discrepancies during the independent data-extraction process. With the assurance of uniformity in the interpretation, the data extraction process was carried out in duplicate for the remaining abstracts.

The data extraction items included the following descriptive information: name of the journal, author addresses including postal address, email or telephone numbers, and medical specialties (e.g. clinical/primary care, surgical/anesthesiology, gynecology/obstetrics, oncology, psychiatry, pediatrics); trial design (e.g., parallel, factorial, superiority, non-inferiority, crossover). Additionally, the following criteria were applied: mentioned randomized or randomly allocated in title, abstract, or both; defined the objective/hypothesis (e.g., to compare the effectiveness of A with B for condition C) or brief background if lacking an objective/hypothesis; identified participant eligibility criteria, settings of data collection, or both; identified interventions intended for each group (intervention and control group); defined primary outcome of the trial; described methods of random sequence generation; described blinding/masking using a generic (simply stating single-blind or double-blind) or detailed (explanation blinding between patients and caregivers, investigators, or outcome assessors) description; provided number of participants randomized and analyzed in each group; identified trial status (whether complete or stopped prematurely); included primary outcome for each group and the estimated effect size with its precision (confidence interval); explained harm reporting; provided conclusions with general interpretation of results or discussed benefit and risks from the intervention; provided information on trial registration and funding. Finally, the abstract format (structured or unstructured) was recorded.

Regarding the assessment of the abstract quality domain, the following characteristics were identified: allocation concealment (simply mentioned or briefly explained the concealment procedures, such as central randomization by telephone or Internet-based system, computer-generated randomization sequence, permuted blocks, sealed envelope with stratification) and blinding (as mentioned above).

### Data analysis

Data for descriptive statistics were analyzed using Microsoft Excel 2007 and the SPSS software (ver. 19.0, IBM SPSS®). We determined the overall number and proportion (%) of RCT abstracts that included each of the items recommended by CONSORT for Abstracts. The kappa statistic was used to measure chance-adjusted inter-observer agreement for reporting aspects of trial quality and significance of results.

## Results

**Figure 1:**
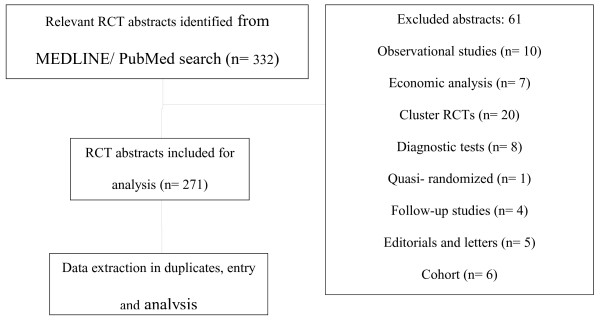
Flow chart of studies considered for inclusion.

Our search strategy identified 332 RTC abstracts from the four high-impact general medical journals during the initial search. Of these, 61 RCT abstracts (10 observational studies, 7 economic analyses, 20 cluster RCTs, 8 diagnostic tests, 1 quasi-randomized trial, 4 follow-up studies, 5 editorials and letters, and 6 cohort studies) were excluded, as shown in Figure 1. Finally, in total, 271 RCT abstracts were selected for the analysis.

### Study characteristics

The numbers of RCTs published in the four high-impact general medical journals are shown in Table 1. Among the 271 included RCT abstracts, 41.7% (113/271) were published in NEJM, followed by Lancet 29.5% (80/271), JAMA 15.9% (43/271), and BMJ 12.9% (35/271). All these journals adopted structured abstract formats: IMRAD (introduction/background, method, results, and discussion) in NEJM and Lancet, and an eight-heading format (objective, design, setting, participants, intervention, main outcome measure, results, and conclusions) in JAMA and BMJ [20].

**Table 1:** Number of RCTs published in four major general medical journals

**Journal name**	**Abstract formats**	Impact factor*	**Number of RCTs identified, *n *(%) (*n*** **= 332)**	**Number of included RCTs, *n *(%) (*n *** **= 271)**
**NEJM**	IMRAD^+^	53.5	128 (38.6)	113 (41.7)
**Lancet**	IMRAD^+^	33.6	92 (27.7)	80 (29.5)
**JAMA**	Eight-heading format^++^	30.0	51 (15.4)	43 (15.9)
**BMJ**	Eight-heading format^++^	13.5	61 (18.4)	35 (12.9)

*JCR 2010 Impact factor; ^+^introduction/background, method, results, and discussion format; ^++^objective, design, setting, participants, intervention, main outcome measure, results, and conclusions format.

### Reporting of general items

Table 2 shows the assessment of CONSORT checklist items reported in the 271 included RCTs. Almost all RCTs (98.9%; 268/271) identified “randomized” in the abstract, but only 58.7% (159/271) reported the same in the title; among these, reporting by NEJM was very low (4.4%; 5/113) compared with the other journals. Of the included abstracts, 70.5% (191/271) provided the authors’ postal and email addresses. Just 23.3% (63/271) of these RCT abstracts provided a description of the trial design (parallel, factorial, crossover, etc.).

**Table 2:** CONSORT checklist items for assessment from abstracts of included RCTs

**Items**	**Assessment criteria**	**Assessment of individual journals, *n *(%)**				**Overall, *n *(%) (*n *** **= 271)**
		**NEJM (*n*** **= 113)**	**Lancet (*n*** **= 80)**	**JAMA (*n*** **= 43)**	**BMJ (*n*** **= 35)**	
**Title**	Study identified as randomized in title	5 (4.4)	79 (98.6)	41 (95.3)	34 (97.1)	159 (58.7)
	Mentioned random/randomized in abstract	110 (97.3)	80 (100)	43 (100)	35 (100)	268 (98.9)
**Authors**	Addresses including postal and emails	75 (66.4)	54 (67.5)	33 (76.7)	29 (82.9)	191 (70.5)
	Only postal address	24 (21.2)	26 (32.5)	7 (16.3)	6 (17.1)	64 (23.6)
**Trial design**	Descriptions provided (parallel, factorial, crossover, etc.)	21 (18.6)	23 (28.8)	9 (20.9)	10 (28.6)	63 (23.3)
**Methods**						
**Participants**	Eligibility criteria with settings of data collection	29 (25.7)	61 (76.3)	40 (93.0)	35 (100)	165 (60.9)
	Only eligibility criteria provided	111 (98.2)	80 (100)	43 (100)	35 (100)	269 (99.3)
**Interventions**	Details including denomination, usage, course of treatment for both groups	97 (85.8)	74 (92.5)	40 (93.0)	35 (100)	246 (90.8)
**Objective**	Specific objective/hypothesis	40 (35.4)	80 (100)	42 (97.7)	35 (100)	197 (72.7)
	Only background described	73 (64.6)	-	1 (0.9)	-	74 (27.3)
**Outcome**	Clearly defined primary outcome	99 (87.6)	80 (100)	43 (100)	35 (100)	257 (94.8)
**Randomization**	Reported the method of random sequence generation	8 (7.1)	71 (88.6)	3 (7.0)	2 (5.7)	84 (31.0)
	Allocation concealment	-	31 (38.6)	-	1 (2.9)	32 (11.8)
**Blinding**	Generic description*	40 (35.4)	26 (32.5)	20 (46.5)	16 (45.7)	102 (37.6)
	Detailed description^++^	2 (1.8)	46 (57.5)	6 (14.0)	3 (8.6)	57 (21.0)
**Results**						
**Numbers randomized**	Number of participants randomized in each group	58 (51.3)	79 (98.6)	32 (74.4)	22 (62.9)	191 (70.5)
**Numbers analyzed**	Number of participants analyzed for each group	58 (51.3)	77 (96.3)	30 (69.8)	21 (60.0)	186 (68.7)
**Outcomes**	Primary outcome result for each group	87 (76.9)	78 (97.5)	39 (90.7)	24 (68.6)	228 (84.1)
	For primary outcome, effect size and confidence interval reported (in total)	35 (31.0)	54 (67.5)	24 (55.8)	15 (42.9)	128 (47.2)
	Effect size and confidence interval (trials with binary outcome)	26 (out of 50) (52.0)	33 (out of 45) (73.3)	12 (out of 19) (63.2)	10 (out of 15) (66.7)	81 (out of 127) (63.8)
	Effect size and confidence interval (trials with continuous outcome)	0 (out of 8)	8 (out of 13)(61.5)	5 (out of 10)(50.0)	2 (out of 6)(33.3)	15 (out of 37) (40.5)
**Harms**	Adverse event or side effect reported	53 (46.9)	50 (62.5)	7 (16.3)	6 (17.1)	116 (42.8)
**Conclusions**	Discussed benefit or harm from the intervention	28 (24.8)	13 (16.3)	5 (11.6)	9 (25.7)	55 (20.3)
	Stated only the benefit from the intervention	84 (74.3)	65 (81.3)	38 (88.4)	25 (71.4)	212 (78.2)
**Trial registration**	Reported registration number and name of trial register	113 (100)	78 (97.5)	43 (100)	35 (100)	269 (99.3)
**Funding**	Reported the source of funding	52 (46.0)	77 (96.3)	-	-	129 (47.6)

*Simply stating single-blind or double-blind; ^++^blinding explained between patients and caregivers, investigators, or outcome assessors.

### Reporting of trial methodology

In total, 60.9% (165/271) of the abstracts reported both eligibility criteria of participants and settings of data collection; among these, NEJM reporting was only 25.7% (29/113). In contrast, 99.3% (269/271) reported only the eligibility criteria of participants. Most of the abstracts (90.8%; 246/271) described details of the intervention including denomination, usage, and course of treatment for both groups. The trial objective/hypothesis was mentioned in 72.7% (197/271), with the remaining 27.3% (74/271) reporting a background or rationale for the study rather than an objective/hypothesis as recommended by CONSORT. This was clearly seen in the NEJM data: only 35.4% (40/113) of abstracts had clear objectives, compared with 97.7% (42/43) in JAMA and 100% in both Lancet (80/80) and BMJ (35/35). Reporting of a clearly defined primary outcome occurred in 94.8% (257/271) of abstracts overall: it occurred in 100% of abstracts in three of the journals but only 87.6% (99/113) of those in NEJM. Only 31% (84/271) of abstracts mentioned the method of random sequence generation, with better reporting in Lancet (88.6%; 71/80) than in the other journals. Kappa values for methods (participants, interventions, objective, outcome, and randomization) were 0.88 or above.

### Reporting of results

From 271 included abstracts, 70.5% (191/271) reported the number of participants randomized to each group, and 68.7% (186/271) reported the number of participants included for analysis (kappa, 0.66 and 0.80). A total of 84.1% (228/271) of abstracts reported the results for the primary outcome measure for each group; however, BMJ reported this value in only 68.6% (24/35) and NEJM 76.9% (87/113), compared with two other journals, which had reporting rates above 90%. The proportion of abstracts describing effect size (ES) and confidence intervals (CI) for the primary outcome was 47.2% (128/271); 63.8% (81 out of 127 trials with binary outcomes) and 40.5% (15 out of 37 trials with continuous outcome) reported both ES and CI. Adverse events or side effects of the intervention were reported in 42.8% (116/271) of abstracts. Inter-observer agreement on abstract reporting of results was 0.86 or above.

### Reporting of conclusions

Regarding the conclusions section of the abstracts, only 20.3% (55/271) stated the benefits and harm from the therapy, in contrast to the 72.8% (212/271) of abstracts that reported only benefits. Overall, most of the RCTs reviewed (99.3%; 269/271) reported that the trial was registered, and 47.6% (129/271) reported funding; however, no abstracts in JAMA or BMJ reported funding. Kappa values for conclusions, trial registration, and funding were 0.95 or above.

### Reporting of trial quality

In total, 11.8% (32/271) of the abstracts mentioned details of allocation concealment, but this information was lacking from abstracts in NEJM and JAMA. With respect to blinding, 37.6% (102/271) mentioned it in generic terms as single blind or double blind, and 21% (57/271) described blinding in detail. Kappa values for quality domains were 0.82 or above.

### Assessing methodology using equal proportion of abstracts per journal

An additional analysis to assess the CONSORT checklist items using an equal proportion of abstracts per journal was carried out. Since BMJ constituted the lowest number of abstracts (*n* = 35), we selected an equal number of RCT abstracts from each journal using a computer-generated simple random sampling method. Table 3 shows the reporting of methodological quality or key features of trial designs and trial results using an equal proportion of abstracts per journal. The details of trial design were included in 21.4% (30/140), reporting of method of random sequence generation in 30.0% (42/140), allocation concealment in 9.3% (13/140), details of blinding in 20.7% (29/140), number of participants analyzed for each group in 69.3% (97/140), and primary outcome result for each group in 82.1% (115/140).

**Table 3:** **Assessment of CONSORT checklist items using an equal proportion of abstracts per journal (*n*** **= 35)**

**Items**	**Assessment criteria**	**Overall, n (%) (*n *** **= 140)**
**Title**	Study identified as randomized in title	104 (74.3)
**Authors**	Addresses including postal and emails	107 (76.4)
**Trial design**	Descriptions provided (parallel, factorial, crossover, etc.)	30 (21.4)
**Participants**	Eligibility criteria with settings of data collection	101 (72.1)
**Interventions**	Details including denomination, usage, course of treatment for both groups	133 (95.0)
**Objective**	Specific objective/hypothesis	118 (84.3)
**Outcome**	Clearly defined primary outcome	137 (97.9)
**Randomization**	Reported the method of random sequence generation	42 (30.0)
	Allocation concealment	13 (9.3)
**Blinding**	Detailed description*	29 (20.7)
**Numbers randomized**	Number of participants randomized in each group	101 (72.1)
**Numbers analyzed**	Number of participants analyzed for each group	97 (69.3)
**Outcomes**	Primary outcome result for each group	115 (82.1)
**Harms**	Adverse event or side effect reported	45 (32.1)
**Conclusions**	Discussed benefit or harm from the intervention	31 (22.1)
**Trial registration**	Reported registration number and name of trial register	139 (99.3)
**Funding**	Reported the source of funding	54 (38.6)

*Blinding explained between patients and caregivers, investigators, or outcome assessors.

## Discussion

We carried out a descriptive cross-sectional study to assess the quality of abstracts for reports of randomized controlled trials from four high-impact general medical journals published in the year 2010, that is, after the release of the CONSORT for Abstracts guidelines in 2008 [7]. Some of the checklist items such as participant eligibility criteria, details of intervention, definition of primary outcome, reporting of primary outcome results for each group, and trial registration were adequately reported. However, harm and funding were reported in fewer than 50.0% of these abstracts. This inadequate reporting is consistent with previous findings for reporting harm [12,21] and funding [21,22]. Two journals, JAMA and BMJ, did not report the source of funding, probably reflecting more of the house style of these journals [13,15].

Contrary to our expectations, the methodological quality domains were still inadequately reported, with no sign of improvement despite the CONSORT recommendations. Table 4 shows a comparison between the assessment of methodological quality domains in the current study and results of a similar study performed previously in relation to the endorsement of CONSORT for abstracts. As compared to other studies, reporting of allocation concealment (11.8%) and details of blinding (21.0%) is higher but still suboptimal in the current study. Abstract reporting is often subject to space constraints and journal formats, which may lead to disparities between full paper results and abstract results. However, a previous study showed that with a word limit of 250–300 words, the checklist items can easily be incorporated [23]. Nevertheless, when an abstract lacks key details about a trial, the assessment of the validity and the applicability of the results become difficult.

**Table 4:** Comparison of methodological quality domains between studies, n (%)

**Studies**	**Allocation concealment**	**Randomization explained**	**Blinding/masking**	
			**Generic**	**Detailed**
**Current study (*n*** **= 271)**	32 (11.8)	84 (31.0)	102 (37.6)	57 (21.0)
**Mann (2011) (*n*** **= 129)**[24]	-	0 (0.0)	-	21 (16.3)
**Wang (2010) (*n*** **= 345)**[22]	0 (0.0)	17 (4.9)	39 (11.3)	1 (0.3)
**Berwanger (2009) (*n*** **= 227)**[12]	1 (0.4)	-	92 (40.5)	21 (9.2)
**Hopewell (2006) (*n*** **= 37)**[25]	0 (0.0)	26 (70.3)	-	6 (16.2)

We also assessed reporting of checklist items for individual journals to compare adherence patterns between journals. Although NEJM and Lancet both adopted the IMRAD abstract format, reporting of the specific objective/hypothesis was minimal in NEJM (35.4%), with only background descriptions in 64.6% of abstracts. NEJM has a very specific style for reporting background rather than objectives [16]; thus, the findings here reflect the specific house style of the journal rather than implementation of the CONSORT for Abstracts guideline. Similarly, for the methodological quality domain, reporting in NEJM and JAMA was 0% for the allocation concealment item, a value comparable to that found in a Chinese study [26]. Funding was also reported in 0% of abstracts in JAMA and BMJ. It has previously been shown that studies funded by pharmaceutical companies have four times the odds of having outcomes favorable to the sponsor than do studies funded by other sources, causing publication bias [27]. Regarding the reporting of conclusions, all four journals failed to provide a balanced summary that noted both benefits and harm from the interventions (11.6%–25.7%), an unsatisfactory value compared with the results from a Chinese study (1.0%) [22]. Between-journal assessments also showed that responses to most of the checklist items were suboptimal for NEJM; these results were consistent with past studies [5,28]. In this inadequate reporting of trials, one positive reporting domain was identified by this study: trial registration, which was reported at a rate of 97.5% by Lancet and 100% by the other three journals. The World Health Organization (WHO), facilitating international collaboration by establishing a clinical trials registry platform [29], and ICMJE’s strict policy of considering trials for publication only if they are registered [30] were likely influences.

Our study has some limitations. Assessment of specialized journals was beyond the scope of this study. Also, we assessed only structured abstracts. Although some studies have suggested that the adoption of structured abstracts by the journals can improve the reporting of trials [31-33], the findings from our study suggest otherwise. The sample of abstracts is influenced by one journal, the NEJM (41.7% of the overall sample); however, a similar trend occurred in 2006 showing the influence of NEJM (41.9%) on overall findings [12]. Moreover, our finding reflects the cross-sectional design. Also, NEJM often published the largest number of RCTs among the other three journals [34]. To minimize the impact of disproportionate abstract samples per journal, we carried out an additional analysis using an equal proportion of abstracts per journal, but the findings were similar with respect to reporting of methodological quality or key features of trial designs and trial results. The analytical strategy known as intention-to-treat (ITT), considered as an optimal approach for preserving the integrity of randomization [35], has been advocated in the full CONSORT 2010 updated guideline [6]; however, this approach has not been incorporated in the CONSORT for Abstracts guideline. Thus, we did not consider inclusion of ITT approach in our assessment. Despite this, our study has several strengths. We reviewed a large number of RCT abstracts published in four high-impact major general medical journals that have universal acceptance among the medical research community. We also conducted an objective data-extraction process, with the domains that were included in the abstracts marked as ‘yes’ and those not reported as ‘no’ based on standard checklist items recommended by the CONSORT group and without reviewer inference. Thus, this study’s methodology is reproducible. Additionally, the period after the CONSORT for Abstracts guidelines were published provides an adequate time frame for the dissemination and endorsement of the CONSORT statement by the research community.

## Conclusions

The findings from our study demonstrate inconsistencies and patterns of non-adherence to the CONSORT for Abstracts guidelines, especially in the methodological quality domains. CONSORT is an evolving guideline [6,36] not considered to be an absolute standard. However, improvements in the quality of RCT reports can be expected by adherence, on the parts both of authors and journal editors during peer review, to existing standards and guidelines as expressed by the CONSORT group. We hope that this will occur in the future.

## Competing interests

The authors declare that they have no competing interests.

## Authors’ contributions

SG and EjK both participated in the design, conduct, assessment, and drafting of the manuscript. EyK and WK conceived the study, interpreted the data, and revised the manuscript. All authors read and approved the final manuscript.
